# A non-human primate model of radiation-induced cachexia

**DOI:** 10.1038/srep23612

**Published:** 2016-03-31

**Authors:** Wanchang Cui, Alexander W. Bennett, Pei Zhang, Kory R. Barrow, Sean R. Kearney, Kim G. Hankey, Cheryl Taylor-Howell, Allison M. Gibbs, Cassandra P. Smith, Thomas J. MacVittie

**Affiliations:** 1 Department of Radiation Oncology, University of Maryland School of Medicine, Baltimore, Maryland, 21201 USA 10 South Pine Street, MSTF Room 604, Baltimore, MD 21201 USA

## Abstract

Cachexia, or muscle wasting, is a serious health threat to victims of radiological accidents or patients receiving radiotherapy. Here, we propose a non-human primate (NHP) radiation-induced cachexia model based on clinical and molecular pathology findings. NHP exposed to potentially lethal partial-body irradiation developed symptoms of cachexia such as body weight loss in a time- and dose-dependent manner. Severe body weight loss as high as 20–25% was observed which was refractory to nutritional intervention. Radiographic imaging indicated that cachectic NHP lost as much as 50% of skeletal muscle. Histological analysis of muscle tissues showed abnormalities such as presence of central nuclei, inflammation, fatty replacement of skeletal muscle, and muscle fiber degeneration. Biochemical parameters such as hemoglobin and albumin levels decreased after radiation exposure. Levels of FBXO32 (Atrogin-1), ActRIIB and myostatin were significantly changed in the irradiated cachectic NHP compared to the non-irradiated NHP. Our data suggest NHP that have been exposed to high dose radiation manifest cachexia-like symptoms in a time- and dose-dependent manner. This model provides a unique opportunity to study the mechanism of radiation-induced cachexia and will aid in efficacy studies of mitigators of this disease.

Cachexia, or muscle wasting, is a serious syndrome associated with many illnesses, including malignant cancer, chronic heart failure (CHF), chronic kidney disease, chronic obstructive pulmonary disease (COPD) and Alzheimer’s disease^1^,^2^. Major hallmarks of cachexia include involuntary loss of body weight (BW) (5% loss in 12 months or less), decreased muscle strength, fatigue, anorexia, low fat-free mass index, and abnormal biochemistry^3^,^4^. It was estimated that more than 50% of cancer patients die with the presence of cachexia, and about 30% of cancer patients die due to cachexia^1^,^2^.

Anticancer therapies, such as chemotherapy and radiotherapy, may induce sequelae such as mucositis, esophagitis, xerostomia, nausea, vomiting and malabsorption, which can lead to anorexia, malnutrition and weight loss^3^,^5^. Clinically relevant losses of BW during radiotherapy in comparison to pre-therapy weights were observed in patients with head and neck cancers^6^,^7^, gastrointestinal cancers^8^ and lung cancers^9^. Cancer treatments may cause BW loss independent of food intake or nutritional supplementation. For example, the weight loss observed in patients receiving concurrent chemo-radiotherapy for non-small cell lung cancer occurred prior to the onset of esophagitis and without decreases in daily nutritional intake^10^.

Victims of radiological accidents may receive very high levels of acute, non-uniform and heterogeneous radiation exposure in contrast to the well-planned and monitored radiation doses administered to patients. The acute radiation syndrome (ARS) is characterized by two major subsyndromes, the hematopoietic (H-) and gastrointestinal (GI-) syndromes (H-ARS, GI-ARS) followed by the delayed effects of acute radiation exposure (DEARE), characterized by multi-organ injury (MOI) that occur in a time- and dose-dependent fashion^11^,^12^,^13^,^14^. Each of these sequelae may be associated with organ-specific morbidity and mortality. Our laboratory has established total-body irradiation (TBI) or partial-body irradiation (PBI) nonhuman primate (NHP) models that permit the study of both short- and long-term damage to the GI, H, lung, heart, kidney and other organ systems. These models were used to study the efficacy of medical countermeasures (MCM) against radiation to enhance survival and overall quality-of-life^15^,^16^,^17^.

Multiple mechanisms are involved in the development of cachexia, including energy imbalance, inflammation, increased protein degradation and decreased protein synthesis, and increased apoptosis^3^,^18^. The ubiquitin-proteasome system (UPS) is the major proteolytic system that degrades proteins in all tissues including muscle^19^,^20^. Activation of the UPS accounts for much of the accelerated muscle proteolysis in many different types of cachectic diseases (cancer cachexia, cardiac heart failure, COPD, etc)^21^. Key players in this pathway include the muscle-specific E3 ubiquitin ligases MuRF1 (TRIM63) and FBXO32 (Atrogin-1). Their induction has been shown to be essential in rapid muscle atrophy^22^. Recently, the myostatin/activin signaling pathway was shown to be critical in triggering muscle wasting in multiple catabolic diseases such as cancer, AIDS, COPD, renal and heart failure^23^. Blocking the myostatin/activin signaling pathway was shown to prevent or reverse loss of skeletal muscle, increase muscle strength and improve survival in various disease models including cancer cachexia and renal failure^24^,^25^,^26^.

In the present study, the BW loss of NHP after high dose radiation exposure was investigated. Combined with other diagnostic factors, including losses in skeletal muscle mass, abnormal blood biochemistry parameters, and abnormal histopathology of muscle tissues, we propose that cachexia is a serious syndrome after high dose radiation exposure. Furthermore, the UPS and myostatin/activin signaling pathways may play important roles in radiation-induced cachexia. Our NHP PBI model may be used as a radiation-induced cachexia model for studying the underlying molecular mechanism and also testing mitigators for this syndrome.

## Results

### Weight loss is the most common indicator for euthanasia after high dose PBI/BM5 exposure

Of the 112 NHP exposed to 9.0 to 12.5 Gy PBI/BM5, 86 (76.8%) were euthanized before the end of the 180 d study period in accordance with IACUC-approved criteria (Table 1). BW loss was one of the most frequent criteria for euthanasia and was observed in 26 of the 112 NHP. Specifically, 17.9% (20/112) euthanasia were performed due to severe BW loss >25% for 4 consecutive days while 5.4% (6/112) were due to BW loss >20% for 4 consecutive days in combination with tachypnea, abnormal activity or appearance, severe dehydration, or edema. Other frequently observed criteria for euthanasia were inactivity (19.6%, 22/112) and respiratory distress or SpO2 <88% (17.9%, 20/112).

Based on the euthanasia criteria in each radiation dose cohort listed in Table 1, the threshold of euthanasia due to BW loss was ∼9.0 Gy. Increased percentage of NHP was euthanized due to BW loss from 9.0 Gy to 11.5 Gy. The percentage was decreased in NHP exposed to 12.0 and 12.5 Gy because these animals were euthanized before severe BW loss could happen (Table 1).

### Changes in BW after exposure to PBI/BM5 is time- and dose-dependent

**Figure 1:**
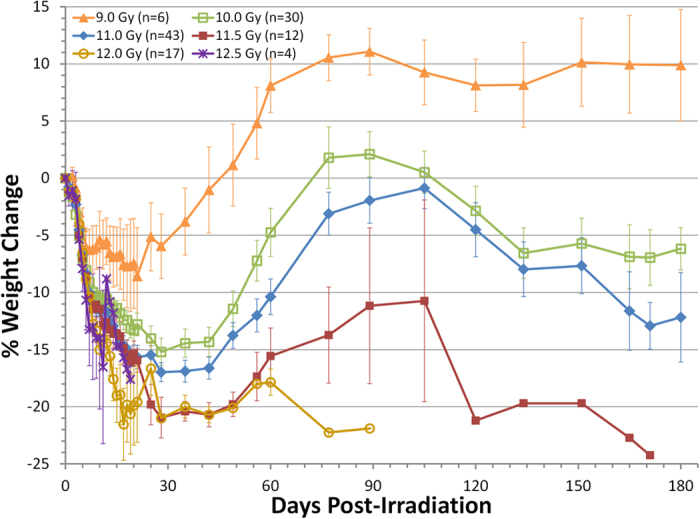
Time course of body weight change of the NHP exposed to 9.0–12.5 Gy PBI/BM5. The % BW change relative to pre-radiation BW is plotted versus days post-irradiation. NHP are grouped according to radiation doses. Mean values (±s.e.m) are displayed for each dose cohort at each time point.

The time courses of BW change in each radiation dose cohort are presented in Fig. 1. All dose cohorts (except the 9 Gy cohorts) exhibited a triphasic pattern consisting of a rapid early post-exposure decrease, a period of weight recovery, and finally a phase of sustained deterioration. Higher doses of radiation were associated with faster and more severe BW loss while lower doses were associated with milder BW loss and better recovery. The NHP exposed to 12.5 Gy and 12.0 Gy exhibited fastest rate of BW loss and a very transient recovery on BW before they were all euthanized. BW of the NHP exposed to 10.0 and 11.5 Gy PBI/BM5 followed a typical triphasic pattern of rapid loss, recovery and deterioration. The NHP exposed to 9.0 Gy exhibited mild loss of BW (<10%) over the first month after exposure followed by recovery of BW that superseded baseline weight after two month.

The initial BW loss was very rapid. In the NHP exposed to 9.0 to 12.5 Gy in the PBI/BM5 model, 90.0% NHP (99/110 alive NHP) lost more than 5% BW at the end of first week after irradiation; and 76.9% NHP (70/91 alive NHP) lost more than 10% BW at the end of two weeks after irradiation. The median BW loss was 9.8% and 12.5% at the end of one and two weeks after irradiation, respectively.

**Figure 2:**
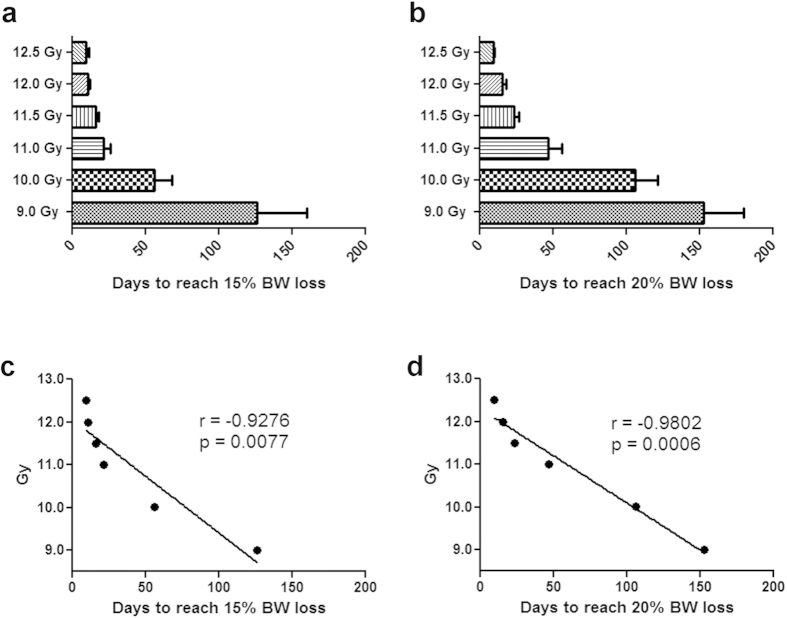
Time to reach 15% or 20% body weight loss at different radiation doses. The time (in days, mean ± s.e.m.) to reach 15% (**a**) or 20% (**b**) body weight loss was plotted for each radiation dose cohort (same animals from Fig. 1). The correlation of the time to reach 15% body weight loss (**c**) or 20% body weight loss (**d**) to radiation doses was plotted. For NHP survived 180 days and didn’t lose 15% or 20% body weight, the time was imputed with 180 because the study was only 180 days. This approach may underestimate the time to reach 15% or 20% body weight loss particularly in the lower radiation dose cohorts.

Time to reach 15% or 20% BW loss in NHP after radiation exposure was radiation dose dependent (Fig. 2). Higher radiation doses were associated with shorter time to reach 15% or 20% BW loss (Fig. 2a,b). Furthermore, the time to reach 15% or 20% BW loss was significantly correlated with radiation doses (Fig. 2c,d).

**Figure 3:**
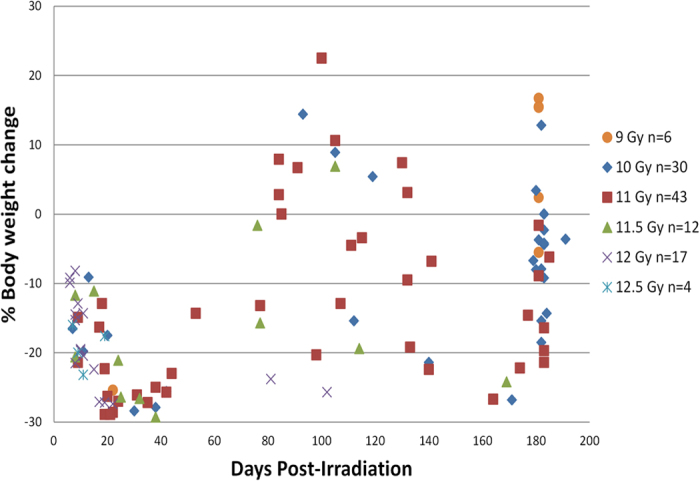
Body weight change at the time of euthanasia of NHP exposed to 9.0–12.5 Gy PBI/BM5. The % BW change of each individual NHP exposed to PBI/BM5 is plotted against the time when the NHP was euthanized. NHP exposed to different radiation doses are marked by different colors and shapes. Each dot represents one NHP.

The unresolved cachectic condition was observed in NHP that were euthanized over the course of the 180 d study duration. Animals were euthanized in accordance with IACUC-approved criteria as indicated above. Unfortunately this precluded our intent to quantify the natural history or mechanism of cachexia in a controlled, longitudinal fashion. However, analysis of BW loss at the time of euthanasia (Fig. 3) provided insight into the evolution of radiation-induced cachexia. Incidents of euthanasia with severe BW loss clustered in two periods: from d 15 to 50 and from d 160 to 180. Few NHP were euthanized because of BW loss between these two periods suggesting that there might be an intermittent attempt to correct the cachectic process after radiation exposure. The timing of severe BW loss after PBI/BM5 exposure suggests that radiation-induced cachexia may also have prodromal, latent, manifest illness, and recovery or death stages similar to those of the ARS.

### Severe loss of lean body mass in NHP exposed to PBI/BM5

**Figure 4:**
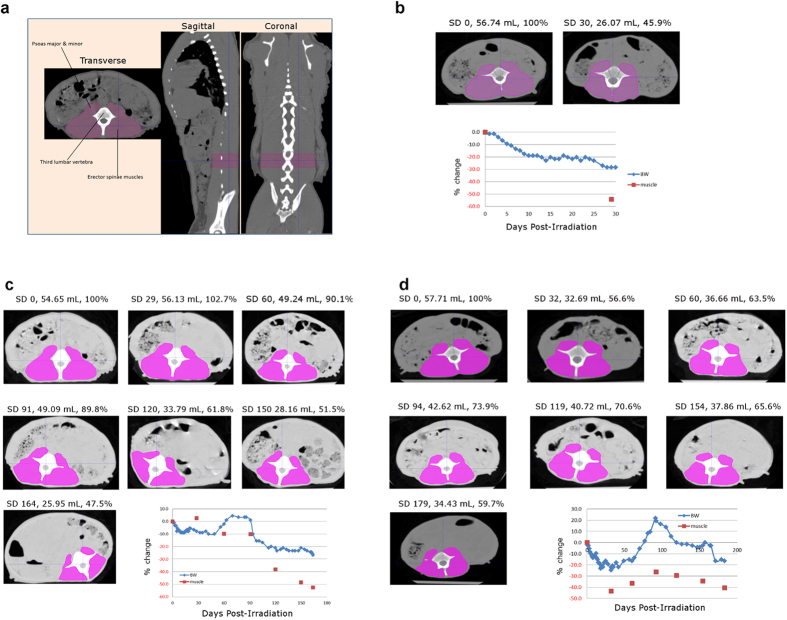
Quantification of skeletal muscle mass using CT imaging. (**a**) Demarcation of skeletal muscle tissues at the 3^rd^ lumbar vertebra. The muscles in this area include the psoas major & minor muscle, and erector spinae muscles. The region was shown by the pink color in three different views. (**b**) CT images of skeletal muscle at the 3^rd^ lumbar vertebra in a NHP exposed to 10 Gy PBI/BM5 which was euthanized on d 30. (**c**) CT images of skeletal muscle at the 3^rd^ lumbar vertebra in a NHP exposed to 10 Gy PBI/BM5 which was euthanized on d 164 after radiation exposure. (**d**) CT images of skeletal muscle at 3^rd^ lumbar vertebra in a NHP exposed to 11 Gy PBI/BM5. This NHP survived the 180 d study after radiation exposure. A graph showing the time course of body weight and skeletal muscle change is included for each NHP. Blue line, body weight change; red line, skeletal muscle change.

Although BW loss is widely recognized as the prominent clinical feature of cachexia, there are many confounding factors (such as edema, obesity, etc.) that may obscure the condition. The loss of skeletal muscle mass is considered the most clinically relevant phenotypic feature of cachexia, irrespective of the underlying causative illness^27^. Analysis of CT images at the 3^rd^ lumbar vertebra has been shown to be a valid approach to quantify total body lean body mass^28^. Therefore, longitudinal CT image analysis on representative animals (One NHP euthanized early due to BW loss, one NHP euthanized late due to BW loss and one survivor) was performed before and every 30 days after PBI/BM5 to study the loss of skeletal muscle. Specifically, CT imaging was utilized to evaluate changes in the volume of the psoas and erector spinae muscles at the third lumbar vertebra (Fig. 4a). As shown in Fig. 4b–d, severe losses of muscle mass appear to parallel the changes in BW after PBI/BM5 but also to a greater extent. The first NHP was exposed to 10 Gy PBI/BM5 and euthanized at d 30 post irradiation, with a BW loss of 28.4% at euthanasia. During the 30 d period, skeletal muscle at the third lumbar vertebra decreased 54.1% from 56.74 mL to 26.07 mL (Fig. 4b). The second NHP was exposed to 10 Gy PBI/BM5 and euthanized at d 164 post exposure. Its BW displayed a decrease, recovery, and decrease triphasic pattern. Its final BW loss was 26.7% at euthanasia. CT image analysis was performed every 30 d after exposure and just prior to euthanasia on d 164. Its skeletal muscle loss was mild from exposure to 3 months after radiation exposure, and then rapidly deteriorated after 3 months. Its skeletal muscle at the third lumbar vertebra decreased 52.5% from 49.09 mL to 25.95 mL at euthanasia (Fig. 4c). The third NHP was exposed to 11 Gy PBI/BM5 and survived the 180 d study, with a typical triphasic BW change following exposure. Its skeletal muscle at the third lumbar vertebra dropped rapidly after radiation exposure, but did show recovery and stabilization after first month. At the end of the study, it had a BW loss of 16.4% and muscle loss of 40.3% (Fig. 4d). The decedent NHP had progressive skeletal muscle loss without recovery, while the surviving NHP had a more stabilized skeletal muscle loss.

### Histological changes in the muscle of irradiated NHP

**Figure 5:**
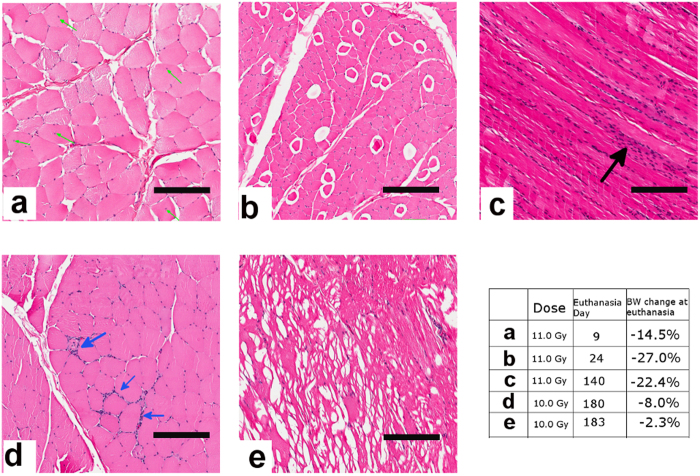
Histological morphology of muscle tissue from five NHP exposed to 10 or 11 Gy PBI/BM5. (**a**) Central nuclei: muscle nuclei (green arrows) are located inside rather than at the periphery of the muscle fibers; (**b**) Adipose replacement of muscle fibers. The hydrophobic fat-rich structures in adipocytes show a clear cytoplasm structure; (**c**) Polynuclei in muscle fibers (black arrows); (**d**) inflammatory cell (blue arrows) infiltration of muscle fibers; and (**e**) branched muscle fibers (left part of the muscle tissue). The table shows radiation doses, time of euthanasia, and BW change at euthanasia compared to pre-irradiation BW for each of the five animals. Scale bar = 100 micron.

Histological abnormalities were observed in the biceps brachii tissues procured from the irradiated NHP at different time points (Fig. 5). Healthy muscle tissue is characterized by polygonal muscle fibers with peripheral nuclei. Muscle fibers with central nuclei were observed in a NHP exposed to 11.0 Gy PBI/BM5 (Fig. 5a), which was euthanized at d 9 with a BW loss of 14.5%. Adipose replacement of myofibrils was observed in a NHP exposed to 11.0 Gy PBI/BM5 and euthanized at d 24 with a 27.0% BW loss (Fig. 5b). In a NHP exposed to 11.0 Gy PBI/BM5, polynuclei were observed in muscle fibers where an abnormally high number of nuclei were detected (Fig. 5c). In a NHP exposed to 10.0 Gy PBI/BM5 and euthanized on d 180 with 8.0% BW loss, inflammatory cell infiltration was observed in muscle fibers (Fig. 5d). Unlike normal skeletal fibers which contain tightly packed protein filaments, branched and torn muscle fibers were observed in a NHP exposed to 10.0Gy PBI/BM5 and euthanized on d 183 with 2.3% BW loss (Fig. 5e). The different histopathologic findings may reflect the different stages in progression of radiation-induced cachexia.

### Decreased serum albumin levels in irradiated NHP

**Figure 6:**
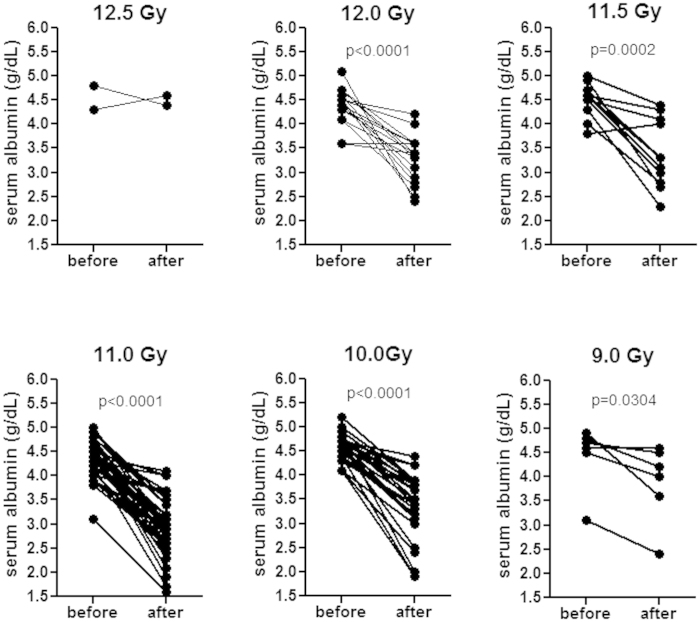
Paired comparisons of serum albumin levels of NHP before and after PBI/BM5. The albumin level after PBI/BM5 is the albumin level on the day of euthanasia or the most recent value if no assay were performed on that day. Note that not all NHP had serum albumin levels measured. Paired comparison of serum albumin levels was performed in each dose cohort. 12.5 Gy (n = 2), 12.0 Gy (n = 17), 11.5 Gy (n = 12), 11.0 Gy (n = 40), 10.0 Gy (n = 27), 9.0 Gy (n = 6). The p value cannot be calculated for the 12.5 Gy because it only had two pairs of data. All other dose cohorts have significantly lower albumin level after PBI/BM5 compared to the baseline level.

Low serum/plasma albumin has been suggested as one criterion for cachexia diagnosis^4^. Serum albumin levels before and after irradiation in NHP exposed to 9.0–12.5 Gy PBI/BM5 were plotted in Fig. 6. Serum albumin levels before irradiation ranged from 3.1 to 5.2 g/dL. Serum albumin levels after irradiation ranged from 1.6 to 4.6 g/dL. All radiation dose cohorts had significantly decreased levels of albumin after PBI/BM5 compared to their baseline levels (except the 12.5 Gy cohorts which didn’t have enough pairs for paired t-test).

### Decreased plasma hemoglobin levels in irradiated NHP

**Figure 7:**
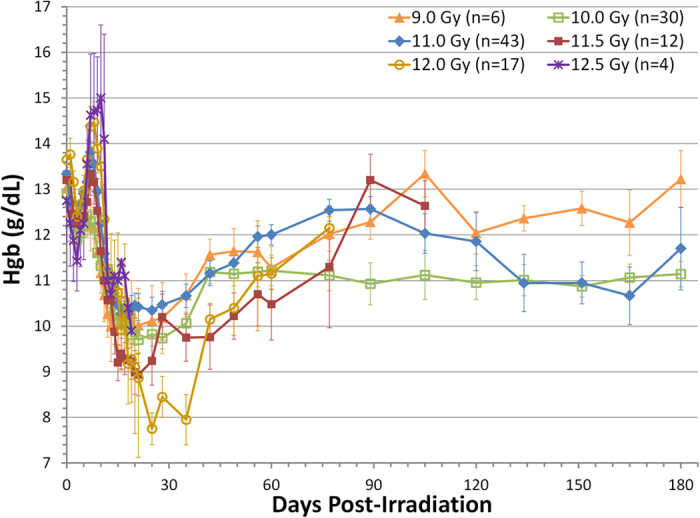
Time course of plasma hemoglobin in NHP exposed to 9.0–12.5 Gy PBI/BM5. Mean values (±s.e.m) are plotted for NHP grouped into each dose cohorts at each time point.

Anemia has been suggested as an additional criterion for cachexia diagnosis^4^. Peripheral blood hemoglobin levels were measured in NHP exposed to 9.0–12.5 Gy PBI/BM5 (Fig. 7). Hemoglobin level was 13.19 ± 0.93 g/dL prior to irradiation. After radiation exposure, hemoglobin levels in all dose groups displayed a multiphase change pattern. The 12.0–12.5 Gy cohort displayed the most drastic change in hemoglobin levels: hemoglobin levels decreased rapidly after exposure till d 3, and then increased around d 7, reached nadir (7.8 g/dL) at around d 25 to 35 (the 12.5 Gy cohort didn’t reach nadir before they were all euthanized), and then slowly recovered, but not to the pre-irradiation level. Other dose cohorts displayed a similar pattern of hemoglobin level change, but to a lesser extent. The high level of hemoglobin at d 5 to 10 may be artificially affected by NHP’s dehydration status at that time. Based on the hemoglobin levels, irradiated NHP were anemic most of the time after 9.0 to 12.5 Gy PBI/BM5.

### Up-regulated UPS pathway in the muscle tissue of irradiated cachectic NHP

**Figure 8:**
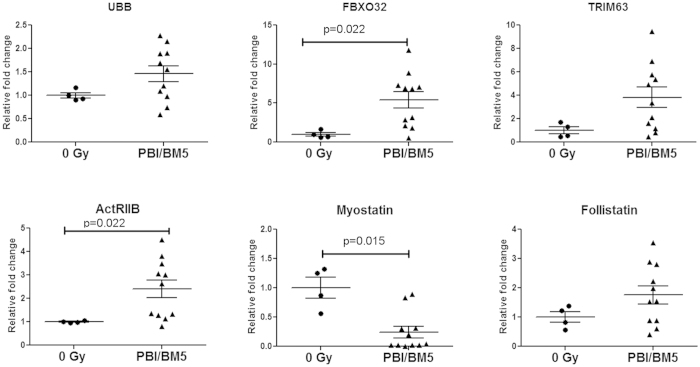
Gene expression change in the irradiated cachectic muscle tissues. mRNA Levels of UBB, FBXO32 (Atrogin-1), TRIM63 (MuRF1), ActRIIB, myostatin and follistatin were quantified in control (0 Gy) and cachectic muscle (BW > 20%) of PBI/BM5 NHP. NHP were exposed to 10 or 11 Gy PBI/BM5. Each symbol represents one animal. n = 4 in the control (0 Gy) group, n = 11 in the PBI/BM5 group. Data are mean ± s.e.m, scatter plot. P value is shown.

To determine whether the UPS pathway was activated in the irradiated cachectic muscle, mRNA levels of UBB, FBXO32, and TRIM63 in the biceps brachii of NHP were measured by q RT-PCR. Each mRNA level in NHP exposed to PBI/BM5 and with >20% BW loss at euthanasia was compared to the non-irradiated control NHP (Fig. 8). The mRNA levels for all three atrophy-related genes in the biceps brachii were increased in the cachectic PBI/BM5 group compared to the control NHP. The relative fold increases (mean ± s.e.m) were 1.46 ± 0.17, 5.39 ± 1.05, and 3.82 ± 0.87 for UBB, FBXO32 and TRIM63 respectively. Particularly, the FBXO32 was significantly different in the PBI/BM5 group compared to the control group (p = 0.022).

### Increased ACTRIIB level and decreased myostatin level in the muscle tissue of irradiated cachectic NHP

To investigate whether the myostatin/activin signaling pathway was also activated in irradiated cachectic NHP, mRNA levels of ActRIIB (ACVR2B), myostatin (MSTN), and follistatin (FST) were measured in the same samples used for examining the UPS pathway activation (Fig. 8). ActRIIB mRNA level was significantly increased (2.40 ± 0.38 fold change, p = 0.022) in the irradiated cachectic group versus the control group. Interestingly, the myostatin mRNA level was significantly decreased in the irradiated cachectic group (0.24 ± 0.10 fold change, p = 0.015) compared to the control group. Follistatin mRNA level was not significantly changed.

## Discussion

The current study showed that high dose radiation exposure caused significant BW loss in a dose-threshold and time-dependent manner in a NHP model. Furthermore, CT imaging analysis provided definitive evidence of skeletal muscle loss, to an extent which exceeded what may be judged from BW change alone. The effect of radiation on skeletal muscle was further supported by histopathologic analysis and quantification of circulating hemoglobin and albumin. Therefore, we propose that cachexia is an important subsyndrome of radiation syndrome. Recognizing this syndrome will help us to further delineate the toxicity of radiation and help to develop mitigators for this syndrome.

There is little information regarding high-level radiation exposure-induced cachexia or muscle wasting. Studies on atomic bomb survivors 50 years after the event showed that atomic bomb radiation was associated significantly and negatively with body mass index and appendicular lean mass, even though the estimated radiation exposures were less than 4 Gy^29^. In the Chernobyl nuclear accident, patients presented with the typical hematological, cutaneous, gastrointestinal syndromes, and neurological/psychological disorders. Little attention has been paid to the patients’ BW change^30^. The under-recognized status of cachexia in victims of high dose radiation exposure may resonate its status in many chronic illnesses^2^. There are possibly many reasons why cachexia has not been a focus in the study of radiation toxicity. Radiation research has been focused on the aforementioned target organs without linking BW loss with cachexia. Additionally, investigation of skeletal muscle loss – the hallmark of cachexia – requires body composition analysis, a technique which may not be widely accessible in the radiation research field. Using such tools in a NHP model, our study examined the occurrence of radiation-induced cachexia in a detailed and thorough manner. With successful management of acute hematological and gastrointestinal syndromes, more attention should be paid to cachexia in victims exposed to high level radiation.

Radiation-induced cachexia may reflect an advanced stage of cachectic disease. The clinical definition of cachexia is usually weight loss of 5% or more over the course of 12 months or less, combined with other criteria such as decreased muscle strength, fatigue, anorexia, low fat-free mass index, and abnormal biochemistry^4^. The weight loss seen here was in a similar range as that seen in advanced cancer and AIDS patients. In pancreatic cancer, 85% of patients were cachectic at diagnosis with a median weight loss of 14.2%; advanced pancreatic or lung cancer and AIDS patients may lose 25–30% of BW shortly before their death^3^. The 50% loss of skeletal muscle in irradiated NHP was also similar to that seen in lung cancer patients, who lose as much as 75% skeletal muscle^3^. One difference between radiation-induced cachexia and chronic disease-associated cachexia is the time course for progression of the weight loss. BW loss in radiation-induced cachexia occurred in an acute time course relative to that noted in other types of cachexia. This rapid progression of the injury suggests that potential mitigators for radiation-induced cachexia should be administered as soon as possible after radiation exposure.

Radiation-induced cachexia may be closely integrated with other radiation subsyndromes. High level radiation is known to cause esophagitis^31^ and acute and prolonged gastrointestinal syndromes^12^,^13^, thus affecting both nutrition intake and absorption. We have shown that intestinal tissue was associated with a prolonged lack of mucosal restitution, crypt regeneration and reconstitution, and a marked increase in goblet cells even 6 months after radiation exposure^13^. Although nutritional support was provided when NHP lost more than 10% BW, it is unknown whether the animals could absorb sufficient nutrients. Therefore, treatment of radiation-induced cachexia will require combination therapy targeting multiple organ systems.

The histopathologic analysis of irradiated muscle tissue suggested that radiation may have various effects on muscles. Nuclei in normal muscle tissue are generally located in the peripheral sides of muscle fibers; myofibers with central nuclei suggest muscle undergoing degeneration/regeneration. Adipose replacement of myofibers is a common feature in many muscular diseases such as Duchenne muscular dystrophy (DMD)^32^, obesity/diabetes^33^, and sarcopenia^34^. The infiltration of inflammatory cells into muscle tissue and torn muscle fibers at 6 month after radiation exposure suggested that radiation has a long lasting effect on skeletal muscles.

The up-regulation of UBB, FBXO32 (atrogin-1) and TRIM63 (MuRF1) in the irradiated and cachectic muscle tissue suggested that radiation-induced cachexia may share a similar pathway to other muscular atrophic conditions. Elevated expression of FBXO32 and TRIM63 were observed in many different models of skeletal muscle atrophy including immobilization, denervation, hindlimb suspension, administration of IL-1, and administration of glucocorticoid dexamethasone^35^. Blocking the UPS pathway may help to mitigate radiation-induced cachexia.

The myostatin/activin signaling pathway was suggested to play a dominant role in the activation of muscle wasting during cancer cachexia^24^. ActRIIB is a high affinity activin type 2 receptor. In the cancer cachexia model, TGF-β family ligands such as myostatin bind to ActRIIB and lead to skeletal and cardiac muscle atrophy. We tested the mRNA levels of ActRIIB, myostatin and follistatin. The elevated ActRIIB level in irradiated cachectic muscle tissue relative to the control group suggested the activity of this pathway may be increased after radiation exposure. Follistatin level was not significantly changed. Interestingly, the myostatin level is decreased in irradiated cachectic muscle tissue compared to the control group. The discrepancy of myostatin mRNA and protein levels in skeletal muscle atrophy^36^,^37^ and the diaphragms of COPD patients^38^ have been reported previously. The data from this study may be in agreement with these findings. The decreased mRNA level of myostatin may be a result of a negative feedback loop with increased myostatin protein negatively regulating its gene expression through a Smad7 dependent mechanism^39^.

In conclusion, we presented data to show that radiation-induced cachexia is a serious sub-syndrome of high level radiation exposure. The decreased BW, decreased lean muscle mass, histopathologic and biochemical data all suggest that cachexia manifested after high level radiation exposure. Radiation-induced cachexia may share similar pathways of protein catabolism and activation of the myostatin/activin signaling as in other cachetic diseases. We presented a NHP model of radiation-induced cachexia that will help to recognize and assess the efficacy of countermeasures for this problem.

## Methods

### Study animals

One hundred and twelve (112) male Chinese rhesus macaques (*Macaca mulatta*) from two contemporary studies were included in this investigation. All the experimental protocols were approved by the University of Maryland Institutional Animal Care and Use Committee (IACUC, Approval Numbers 0409011 and 0312017). The primary endpoint for these studies was survival at 180 days (d) post radiation exposure. NHP housing and care was performed in accordance with the Animal Welfare Act (7 U.S.C. 2131 et seq.) at the University of Maryland’s AAALAC-accredited animal facility. NHP were provided certified primate chow (Harlan Teklad) *ad libitum* that was supplemented with fresh fruit and vegetables (e.g., apples, bananas, pears, grapes, sweet potato, green beans) and primate treats. All NHP enrolled in the studies were in good health, sero-negative for simian immunodeficiency virus, simian T cell leukemia virus type 1, and herpes B virus, and negative for malaria and tuberculosis.

The current paper is a retrospective study on animals from previous studies. Therefore, clinical data and tissue samples may not be available for each animal. For example, albumin level was not measured for each animal. Muscle tissue was not collected for each animal. The authors tried the best to use the maximum number of available data and samples.

### Irradiation and dosimetry

NHP were exposed to 9.0 to 12.5 Gy partial-body irradiation with 5% bone marrow sparing (PBI/BM5) according to an Institutional Animal Care and Use Committee (IACUC)-approved protocol, as described previously^11^,^13^. Irradiations were performed at the University of Maryland Medical Center, Department of Radiation Oncology, utilizing 6 MV LINAC-derived photons at a dose rate of approximately 0.80 Gy/min (2 MV average, Varian TrueBeam™). NHP were sedated with ketamine, secured in a supine restraint device and administered xylazine to ensure proper radiation field placement would be sustained. At irradiation, NHP were positioned with their tibiae, ankles, and feet outside of the beam field to spare approximately 5% of the bone marrow. Radiation doses were prescribed to each animal’s midline as measured by the separation at the xiphoid process. Exposures were bilateral and uniform, with half of each prescribed dose delivered by an anteroposterior (AP) beam, and half by a posteroanterior (PA) beam. *In vivo* dosimetry was performed using either silicon diodes or optically stimulated luminescence detectors (OSLDs, Landauer nanoDots™), which were placed on the chest to measure the delivered doses. Dosimeters were also placed on the anatomical areas to be spared, to confirm that they were not included in the exposure field.

### Medical management

All NHP received medical management (aka supportive care) during the study according to an IACUC approved protocol of triggers and treatments, as described previously^11^,^13^. Treatments included hydration fluids, antibiotics, analgesics, antidiarrheals, antipyretics, antiemetics, cytoprotection for mucosal ulcers, corticosteroids, nutritional support, and blood transfusions. To manage loss of BW, animals that lost ≥10% of their pre-irradiation BW were administered either BIO-SERV certified Rhesus “Liquidiets” or High Protein Shake (HPS) containing a mixture of medium chain triglyceride (MCT) oil and protein supplement liquid (Pro-Mod^®^, Abbot Nutrition) at 15 mL/kg/d by oral gavage. The volume of “Liquidiets” or HPS was reduced to 7 mL/kg/d or 10 mL/kg/d if the animal was also receiving treated water or oral electrolyte solution respectively. Cage-side observations were performed twice per d. The animals were observed for 180 d post-exposure, or until euthanasia per IACUC-stipulated clinical criteria. All medical management procedures were performed in accordance with the Animal Welfare Act.

Among the 112 NHP in this study, 38 NHP received Neupogen (filgrastim) dosed at 10 μg/kg/d starting at d 1, d 3, or d 5 post irradiation to study its effect on survival. All other NHP receive 5% Dextrose in water or no treatment. For the purpose of the current study, the effect of administration of Neupogen on cachexia was not considered.

### Euthanasia

Detailed euthanasia criteria were described previously^13^. Euthanasia criteria were specified according to an IACUC-approved protocol, which include persistent loss of BW, hyperthermia or hypothermia, self-mutilation, evidence of unrelieved pain or distress, seizure activity, inactivity, excessive hemorrhage, or respiratory distress. NHP were euthanized with DEA Class III euthanasia solution (Euthasol^®^, Vibrac AH Inc., Fort Worth, TX). All surviving animals were euthanized at the end of each study.

### Computed tomography (CT) scans

Non-contrast CT scans were performed at selected time points to evaluate skeletal muscle loss using a Lightspeed™ CT scanner (General Electric Healthcare, Waukesha, WI). The radiographic analysis was performed on a workstation running image analysis software (MIM Maestro™ Software, MIM software, Inc., Cleveland, OH). Muscle mass quantification was based on the method described by Baracos V^28^. Briefly, 3-D images of skeletal muscle at the third lumbar vertebra were demarcated and the volume of the skeletal muscle in the demarcated region was calculated. The region contains *psoas major* and *minor* muscles, as well as *erector spinae* muscles. CT scans were performed before irradiation, every month after irradiation, and prior to euthanasia when possible.

### Muscle tissue collection

Biceps brachii samples were collected at necropsy and stored in RNAlater^®^ (QIAGEN Inc., Valencia, CA) or formalin solution for molecular and histological studies. Tissues from four non-irradiated control NHP were included for the molecular analysis.

### Histological study

Hematoxylin and eosin (H&E) staining was performed on available biceps brachii tissues using a standard procedure on formalin-fixed and paraffin-embedded tissue sections.

### Real-time quantitative reverse transcription PCR (qRT-PCR)

Total RNA was isolated from homogenized samples of muscle tissue using Qiagen’s RNeasy Fibrous Tissue Mini kit with DNase I treatment. Total RNA was reverse transcribed into cDNA using iScript™ Reverse Transcription Supermix for RT-qPCR (Bio-Rad Laboratories Inc., Hercules, CA). The real-time PCR reaction included SsoFast Probes supermix (Bio-Rad Laboratories Inc.), *Macaca mulatta* specific Taqman^®^ Gene probes (Life Technologies, Frederick, MD) and cDNA template. The probes include TRIM63 (Rh00822399_m1), FBXO32 (Rh02852340), UBB (Rh02795674_g1), ACVR2B (Rh00609605_m1), FST(Rh01121165_m1), MSTN (Rh02857249_m1) and Actin (Rh03043379_gH). The assay was performed on the CFX96 Real-time PCR Detection System (Bio-Rad Laboratories Inc.). Each sample was run in duplicate, individual results for each target were normalized against their respective endogenous control (Actin), and values for each sample were reported as fold change relative to that of the control NHP.

### Serum albumin measurement

Serum albumin levels were determined using an ACE^®^ Clinical Chemistry System (Alfa Wassermann Diagnostic Tech, LLC, West Caldwell, NJ).

### Plasma hemoglobin measurement

Plasma hemoglobin levels were determined using a Beckman Coulter Ac∙T diff or Ac∙T diff2 Analyzer (Beckman Coulter, Inc. 11800 SW 147^th^ Ave, Miami, FL).

### Statistical analysis

All data were expressed as the mean ± s.e.m. Differences of the albumin levels before and after radiation were compared using two tailed paired t-test since the values had normal distribution. Differences of the realtime PCR results were analyzed using two tailed Mann-Whitney t-test because the sample size was small in the 0 Gy group. Correlations were calculated using Pearson’s correlation analysis. A p-value <0.05 was considered significantly different.

## Additional Information

**How to cite this article**: Cui, W. *et al.* A non-human primate model of radiation-induced cachexia. *Sci. Rep.* **6**, 23612; doi: 10.1038/srep23612 (2016).

## Figures and Tables

**Table 1:** Profile of the euthanasia criteria met for NHP exposed to 9.0–12.5 Gy PBI/BM5 before the end of study at 180 days post-irradiation.

Radiation Doses (Gy)	Total # of NHP	Number (%) of NHP euthanized before end of study	Number (%) of NHP euthanized before end of study for criteria				
			25% BW loss >72h	20% BW loss >72h combination	Inactivity	Respiratory distress or SpO_2 _< 88%	Others
9.0	6	1 (16.7)	0	1 (16.7)	0	0	0
10.0	30	15 (50.0)	5 (16.7)	0	4 (13.3)	5 (16.7)	1 (6.7)
11.0	43	37 (86.0)	8 (18.6)	2 (4.6)	6 (14.0)	11 (25.6)	10 (23.3)
11.5	12	12 (100)	4 (33.3)	1 (8.3)	2 (16.7)	3 (25.0)	3 (25.0)
12.0	17	17 (100)	3 (17.6)	2 (11.8)	8 (47.1)	0	4 (23.5)
12.5	4	4 (100)	0	0	2 (50.0)	1 (25.0)	1 (25.0)
All doses	112	86 (76.8)	20 (17.9)	6 (5.4)	22 (19.6)	20 (17.9)	19 (17.0)
